# Supplementary material for: Analysis of the Association Between MicroRNA Biogenesis Gene Polymorphisms and Venous Thromboembolism in Koreans
Source: Int J Mol Sci. 2019 Aug 1;20(15):3771. doi: 10.3390/ijms20153771 (PMC6695971; doi:10.3390/ijms20153771)
Supplement: Supplementary file 1 [file ijms-20-03771-s001.pdf]

Supplementary Table 1. List of miRNAs that depend on the level of miRNA biogenesis gene expression.

Biogenesis gene	miRNA	Reference
DICER1	miR-21, miR-22, miR-100, miR-221, miR-ler-7d, miR-130a, miR-17	[24]
	miR-222-3p, miR-21-3p, miR-21a,3p, miR-27a-3p	[23]
DROSHA	miR-10a, miR-10b, miR-218, miR-25, miR-19a, miR-26a	[25]
	miR-16-5p, miR-21-5p	[23]
RAN	miR-103a, miR-182, miR-191, miR-223	[26]
XPO5	miR-200c-3p, miR-141-3p, miR-141-5p	[23]
	miR-302c, miR-125b, miR-196a, miR-155, miR-527, miR-300b, miR-1060	[27]

Supplementary Table 2. PCR-RFLP conditions for detection of miRNA biogenesis genes polymorphisms.

SNP	Ref. gene	Polymorphism	Forward primer (5′-3′)	Reverse primer (5′-3′)	Restriction enzyme[a]
rs3742330	*DICER1*	A>G	5'- GGT CTC AGT TTG GTG GCT TC -3'	5'- CCT GCC TTG ACA ACA TGA AA -3'	*Ban*II
rs10719	*DROSHA*	T>C	5'-CTA GTT TTC CTG CAG ACA ATG CA-3'	5'-GTA ATG CAC ATT CAC CAA AGT CA-3'	*Dra*III
rs14035	*RAN*	C>T	5'-GAA GCA CTT GCT CAA AAT CTG TGA C-3'	5'- TGC CAT CCA CTG ATG TTC CAT C-3'	*Bsl*I
rs11077	*XPO5*	A>C	5'-TGC TTT GGG CAA GAA TCT GGT CAC-3'	5'-TAA AGG GGA TGT TAG CAC TAA AGA AT -3'	*Bsm*I

[a]All restriction enzymes were obtained from New England Biolabs (Ipswich, MA, USA), and the manufacturer recommended reaction conditions were used.
Abbreviations: PCR-RFLP, polymerase chain reaction-restriction fragment length polymorphism, SNP, single-nucleotide polymorphism.
